# Luteolin Treatment Protects against Renal Ischemia-Reperfusion Injury in Rats

**DOI:** 10.1155/2017/9783893

**Published:** 2017-11-21

**Authors:** Xin Hong, Xiaojing Zhao, Gang Wang, Zhengliang Zhang, Honghong Pei, Zhong Liu

**Affiliations:** Department of Emergency Medicine, The Second Affiliated Hospital of Xi'an Jiaotong University, Xi'an, Shaanxi 710004, China

## Abstract

Renal ischemia-reperfusion (I/R) injury is a common but severe scientific problem. Luteolin has great anti-inflammatory and antioxidant effects. In this study, we studied the effect of luteolin on renal I/R injury in rats. Intragastric administration of luteolin or saline was performed in Sprague-Dawley rats before (40 mg/kg for three days) and after (one day) renal I/R modeling. Kidney and blood samples were harvested to detect the severity of renal injury 24 hours after operation. The results showed that luteolin-treated rats exhibited milder histomorphological changes with lower scores of renal histological lesions; lower blood urea nitrogen and creatinine levels; lower renal malondialdehyde (MDA), 8-oxo-deoxyguanosine (8-OHdG), and myeloperoxidase (MPO) levels; and higher superoxide dismutase (SOD) and catalase (CAT) activities in the kidney. Luteolin attenuated the increased levels of serum and renal tumor necrosis factor (TNF)-*α*, interleukin (IL)-1*β*, and IL-6, renal high mobility group box-1 (HMGB1), and nuclear factor kappa *β* (NF-*κ*B) expression levels in I/R rats. Furthermore, luteolin treatment significantly reduced renal cell apoptosis and endoplasmic reticulum (ER) stress caused by renal I/R injury. In conclusion, luteolin improved renal function in I/R rats by reducing oxidative stress, neutrophil infiltration, inflammation, renal cell apoptosis, and expression of HMGB1 and NF-*κ*B, and ER stress.

## 1. Introduction

Renal ischemia-reperfusion (I/R) injury is mainly induced by surgery requiring clamping of the aorta, renovascular surgery, shock, trauma, and renal transplantation, which is the most frequent cause of acute kidney injury (AKI) [1, 2]. In particular, in renal transplantation, the I/R damage could cause graft dysfunction and rejection, resulting in severe postoperative complications and death [3]. Despite many efforts have been done, the pathophysiology and exact mechanisms of I/R-induced renal injury are still not well illustrated. Dysfunction of tubular epithelial cells, microcirculatory disorders, robust inflammatory reaction, loss of endothelial integrity, activation of neutrophils, and release of reactive oxygen species (ROS) are generally accepted pathologic processes that all play important roles in I/R-induced renal injury. The methods applied for the attenuation of renal I/R injury include various anti-inflammatory and antioxidant drugs, endocrine hormones, erythropoietin, small interfering RNA, and others [4]. However, various drawbacks have prevented their clinical application. It is important to explore new and effective methods to decrease renal I/R injury to solve this problem.

Luteolin (3′,4′,5,7-tetrahydroxyflavone) is abundant in vegetables, fruits, and plants [5]. Basic and clinical studies show that luteolin has major biological properties, including antioxidant, antiapoptosis, and anti-inflammation effects. Therapeutic applications of luteolin have been reported in some chronic inflammatory diseases, atherosclerosis, drug-induced liver injury, diabetes, cancer treatment, antibacterial therapy, and so forth [6]. In regard to its effect in renal diseases, luteolin can protect against colistin/cisplatin-induced nephrotoxicity, diabetic nephropathy, lipopolysaccharide- (LPS-) induced acute renal injury, and so forth [7–9]. However, the role of luteolin treatment on I/R-induced renal damage has not been explored. The aim of the present study was to explore the effect and mechanism of luteolin in reducing ROS, inflammation, apoptosis, and endoplasmic reticulum (ER) stress in the kidney of rats caused by renal I/R damage.

## 2. Materials and Methods

### 2.1. Experimental Rats and Reagents

Adult male Sprague-Dawley rats (210–250 g, Animal feeding center of Xi'an Jiaotong University Health Science Center) were used in the present study. The experimental rats were fed a conventional and standard rat chow and clean water under a 12 h light-dark cycle. This experiment was approved by and cared for in accordance with the ethical committee, Xi'an Jiaotong University Health Science Center. The drug of luteolin was purchased and from Sigma-Aldrich Co. LLC. (Sigma-Aldrich, St Louis, MO).

### 2.2. Renal I/R Modeling

Intraperitoneal injection (i.p) of chloral hydrate (10%, 250 mg/kg) was used to anesthetize the rats. After middle laparotomy, right nephrectomy was performed first. Then, the left renal pedicle was clamped by the microaneurysm clamp. The microaneurysm clamp was removed 45 minutes after clamping, and the rat was monitored alive. The abnormal incision was sutured in 2 layers [10]. The rats received 1 ml saline subcutaneous injection for recovery from anesthesia and were allowed free chow and clean water after the operation.

### 2.3. Experimental Groups

**Figure 1:**
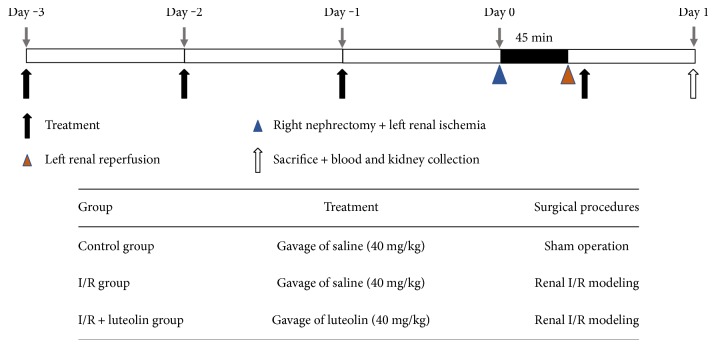
The experimental schematic of the study.

The rats were randomly divided into the following three groups: (1) sham control group: after laparotomy, the bilateral kidneys were isolated from the surrounding tissues and replaced gently without resection; (2) saline-treated I/R group (I/R group): saline was given orally before (40 mg/kg for three days) and after (40 mg/kg for one day) renal I/R experimentation; and (3) luteolin-treated I/R group (I/R + luteolin group): luteolin (40 mg/kg/day) was given orally before (40 mg/kg for three days) and after (40 mg/kg for one day) renal I/R experimentation. The rats (*n* = 6 in each group) in these experimental groups were sacrificed 24 hours after surgery to determine the degree of renal injury. The experimental schematic is presented in Figure 1. Kidney and blood samples were harvested for further detection. Serum samples were separated from blood samples by centrifugation at 4°C and 3000 ×g for 15 min.

### 2.4. Sample Collection and Renal Function Measurement

Serum levels of blood creatinine (Cr) and urea nitrogen (BUN) were detected as the index of renal function in the clinical laboratory, The Second Affiliated Hospital, Xi'an Jiaotong University. Renal tissues were removed and fixed by 10% buffered formalin and embedded 24 hours later in paraffin for histopathologic examination. The rest of the kidney specimen was snap frozen by liquid nitrogen and kept at −80°C for biochemical analyses.

### 2.5. Histological Study and Renal Injury Scoring

Serial sections of the paraffin-embedded kidney (5 *μ*m thickness) were made and treated with hematoxylin and eosin (H&E) staining to assess the pathologic changes. Renal injury scores were determined by two researchers in a blinded fashion according to the extent of kidney injury, as previously described. Briefly, the scoring grading was mainly based on the hemorrhage, tubular cell necrosis, tubular dilatation, and cytoplasmic vacuole formation. The grading system was shown as the following scoring: 0 (normal kidney); 1 (0–5% injury, minimal damage); 2 (5–25% injury, mild damage); 3 (25–75% injury, moderate damage); and 4 (75–100% injury, severe damage) [11].

### 2.6. Apoptosis Assessment

Serial sections of paraffin-embedded kidneys (4 *μ*m thickness) were used to perform the terminal deoxynucleotidyl transferase-mediated nick end labeling apoptosis assay (TUNEL). 4′,6-diamidino-2-phenylindole (DAPI) was used to label the nucleus (blue), and TUNEL assays were performed to detect the apoptotic renal cells (green). The results were observed using a fluorescence microscope. The apoptotic cells were calculated, as previously described [12].

### 2.7. Cytokine Measurement in Murine Serum and Kidney

Serum and renal tissue levels of TNF-*α*, IL-1*β*, and IL-6 were assessed by relevant commercial ELISA kits and performed according to the instructions (Beyotime Biotechnology, Shanghai, China).

### 2.8. Oxidative Stress Measurement in Murine Kidney

Renal tissue homogenate (10%, *w*/*v* in saline) was prepared and centrifuged at 4°C and 4000 ×g for 30 min to collect the supernatants. Malondialdehyde (MDA), 8-hydroxy-2′-deoxyguanosine (8-OHdG), superoxide dismutase (SOD), catalase (CAT), and myeloperoxidase (MPO) activities in the supernatants were measured by using the activity assay kits and ELISA kits according to the manufacturer's instructions (Nanjing Jiancheng Bioengineering Institute and Beyotime Biotechnology).

### 2.9. Immunohistochemistry (IHC)

For HMGB1 and NF-*κ*B localizations and semiquantitation, serial sections of the paraffin-embedded kidney (4 *μ*m thickness) were mounted onto saline-coated slides, dewaxed, rehydrated in a graded series of alcohol, and rinsed in distilled water. The polyclonal antibodies of HMGB1 (1 : 50) and NF-*κ*B (1 : 100) (Biosynthesis Biotechnology Co., Ltd, Beijing, China) were used. The semiquantitative of HMGB1 and NF-*κ*B expressions was calculated according to the intensity and extent of IHC staining. The intensity grading system: 0, negative; 1, weak; 2, moderate strong; and 3, strong. The extent grading system basing on the positive cells staining: 0, negative; 1, 1%–25%; 2, 26%–50%; 3, 51%–75%; and 4, 76%–100%. The staining score was the mean of the sum of the intensity and extent scoring from six fields ranging from 0–12.

### 2.10. Western Blotting Analysis

The renal proteins were loaded and separated on 8% polyacrylamide gel and transferred to polyvinylidene difluoride membranes. The membranes were blocked overnight with 5% (*w*/*v*) skimmed milk and probed with goat anti-C/EBP homologous protein (CHOP), 78 kDa glucose-regulated protein (GRP78), activating transcription factor 4 (ATF4), spliced X-box binding protein1 (XBP1s) (Biosynthesis Biotechnology Co., Ltd, Beijing, China), and rabbit anti-actin antibodies at 37°C for 2.5 hours. Subsequently, the membranes were washed and further incubated with anti-rabbit or anti-goat IgG for 2 hours. The diaminobenzidine method was used to detect positive bands. The blots were analyzed and calculated by ImageJ software (https://imagej.nih.gov/ij/).

### 2.11. Statistical Analyses

GraphPad Prism 6.0 software (version 6.0, GraphPad Software, Inc., La Jolla, CA, USA) was used for statistical analysis. All data were expressed and presented as mean and standard error of mean (mean ± SEM). Statistical differences between two or multiple groups were analyzed by Student's *t*-test or one-way ANOVA. Significant differences were determined by *P* < 0.05.

## 3. Results

### 3.1. Luteolin Treatment Was Effective in Alleviating Renal I/R Injury

**Figure 2:**
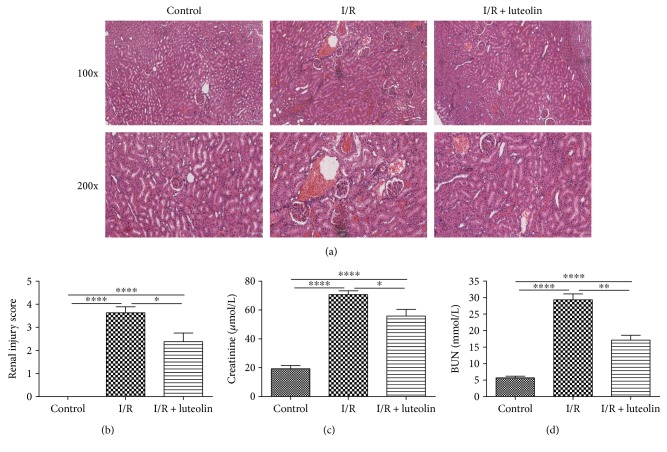
Luteolin treatment reduced the renal ischemia-reperfusion (I/R) injury in rats 24 hours after reperfusion. Renal tissues and blood samples from control, I/R, and I/R + luteolin group rats were collected 24 hours after reperfusion. (a) Representative photomicrographs of renal tissues stained by hematoxylin and eosin at 100 and 200 magnification, (b) renal injury score, (c) serum creatinine, and (d) serum BUN were adopted to detect the protective effect of luteolin on renal I/R injury. All data are expressed as the mean ± SEM, *n* = 6. ^∗^*P* < 0.05, ^∗∗^*P* < 0.01, and ^∗∗∗∗^*P* < 0.0001.

The H&E staining results showed that the kidneys from the I/R group rats displayed hemorrhage, detachment and swelling of the tubular epithelial cells, interstitial edema, tubular cell casts and dilatation, and necrosis. However, luteolin treatment decreased the impaired histopathology and preserved the normal morphology of the kidney, showing slight edema of the tubular cells and less necrosis (Figure 2(a)). The renal injury scoring (I/R group versus I/R + luteolin group, 3.625 ± 0.2631 versus 2.375 ± 0.3750, *P* = 0.0163, Figure 2(b)) showed that luteolin treatment could significantly decrease the renal I/R injury, which was consistent with the H&E results.

### 3.2. Luteolin Treatment Was Effective in Improving Renal Function

Both the Cr and BUN were significantly increased 24 hours after the renal I/R operation in the I/R group when compared with the control group (I/R group versus control group, Cr: 70.67 ± 2.712 versus 19.33 ± 2.246 *μ*mol/L, *P* < 0.0001; I/R group versus control group, BUN: 29.31 ± 1.832 versus 5.700 ± 0.4868 mmol/L, *P* < 0.0001). Luteolin treatment resulted in significantly reduced serum levels of Cr and BUN (I/R group versus I/R + luteolin group, Cr: 55.87 ± 4.545 versus 70.67 ± 2.712 *μ*mol/L, *P* = 0.0189; I/R group versus I/R + luteolin group, BUN: 17.14 ± 1.463 versus 29.31 ± 1.832 mmol/L, *P* = 0.0058) at 24 hours after reperfusion (Figures 2(c) and 2(d)).

### 3.3. Luteolin Treatment Was Effective in Inhibiting Oxidative Stress and Improving the Antioxidant Enzymatic Activities

**Figure 3:**
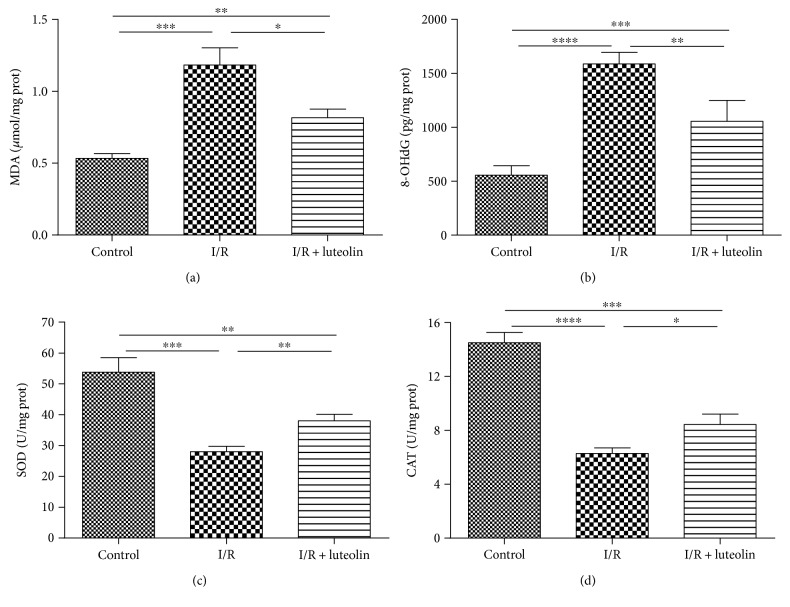
Luteolin treatment attenuated oxidative stress and increased antioxidant ability in rats 24 hours after reperfusion. Renal tissues from control, I/R, and I/R + luteolin group rats were collected 24 hours after reperfusion. Markers of oxidative stress including (a) MDA and (b) 8-OHdG and markers of antioxidant ability including (c) SOD and (d) CAT were detected using the activity assay and ELISA kits. All data are expressed as the mean ± SEM, *n* = 6. ^∗^*P* < 0.05, ^∗∗^*P* < 0.01, ^∗∗∗^*P* < 0.001, and ^∗∗∗∗^*P* < 0.0001.

A balance between promoting and suppressing oxidative stress is associated with I/R-induced renal injury. Oxidant stress was assessed by detecting the MDA and 8-OHdG levels in the kidney 24 hours after experimentation. The treatment of luteolin could significantly decrease the renal MDA and 8-OHdG levels in comparison to saline treatment in the I/R group (Figures 3(a) and 3(b)). Luteolin treatment significantly improved the SOD and CAT activities compared with the I/R group (Figures 3(c) and 3(d)).

### 3.4. Luteolin Treatment Was Effective in Inhibiting Neutrophil Infiltration

**Figure 4:**
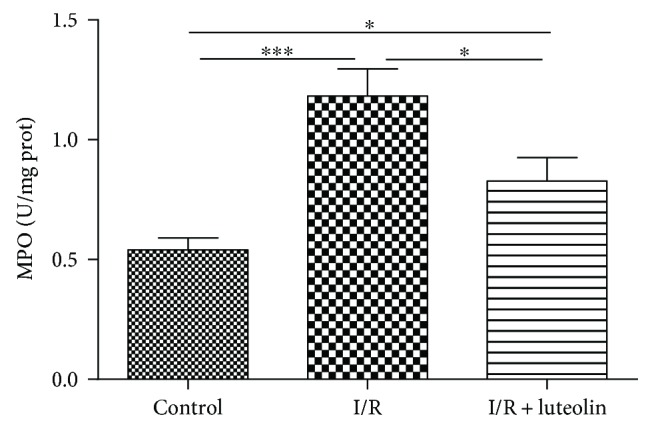
Luteolin treatment decreased neutrophil infiltration in rats 24 hours after reperfusion. Renal tissues from control, I/R, and I/R + luteolin group rats were collected 24 hours after reperfusion. A marker of neutrophil infiltration (MPO) was measured using the activity assay kits. All data are expressed as the mean ± SEM, *n* = 6. ^∗^*P* < 0.05 and ^∗∗∗^*P* < 0.001.

The MPO activity was measured 24 hours after the experiment to reflect the neutrophil infiltration and accumulation in the kidney. When compared with saline treatment in the I/R group, luteolin treatment significantly decreased the MPO activity and neutrophil infiltration (Figure 4).

### 3.5. Luteolin Treatment Was Effective in Inhibiting Inflammation

**Figure 5:**
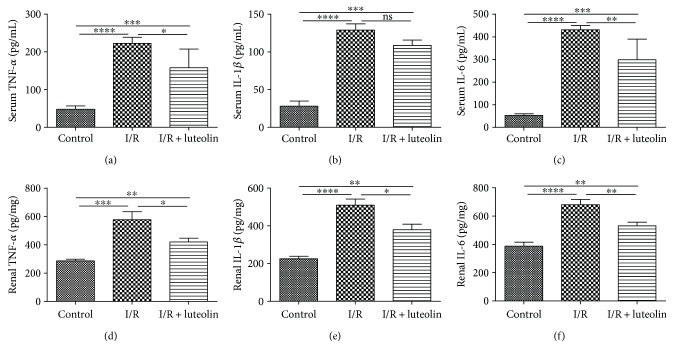
Luteolin treatment inhibited inflammation in rats 24 hours after reperfusion. Renal tissues from control, I/R, and I/R + luteolin group rats were collected 24 hours after reperfusion. Proinflammatory cytokines including (a) TNF-*α*, (b) IL-1*β*, and (c) IL-6 were detected by ELISA kits. All data are expressed as the mean ± SEM, *n* = 6. ^∗^*P* < 0.05, ^∗∗^*P* < 0.01, ^∗∗∗^*P* < 0.001, and ^∗∗∗∗^*P* < 0.0001. ns: no significance.

The serum and renal levels of TNF-*α*, IL-1*β*, and IL-6 were significantly elevated after renal ischemia compared with the control group. However, luteolin treatment could significantly decrease both the serum and renal levels of TNF-*α*, IL-1*β*, and IL-6 in comparison to the I/R group (Figure 5).

### 3.6. Luteolin Treatment Was Effective in Inhibiting NF-*κ*B and HMGB1 Expressions

**Figure 6:**
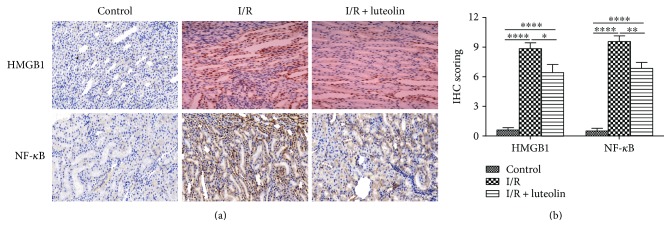
Luteolin treatment inhibited HMGB1 and NF-*κ*B expression in rats 24 hours after reperfusion. Renal tissues from control, I/R, and I/R + luteolin group rats were collected and subjected to immunohistochemical staining (IHC) 24 hours after reperfusion. (a) Representative photographs of HMGB1 and NF-*κ*B expression levels in the renal tissues (original magnifications, ×200); (b) the IHC scoring was calculated. All data are expressed as the mean ± SEM, *n* = 6. ^∗^*P* < 0.05, ^∗∗^*P* < 0.01, and ^∗∗∗∗^*P* < 0.0001.

The inflammation associated proteins HMGB1 and NF-*κ*B were detected by IHC staining. The results showed that HMGB1 and NF-*κ*B staining in the kidney were significantly increased after the I/R operation. However, luteolin treatment could significantly decrease the HMGB1 and NF-*κ*B staining (Figure 6(a)). For semiquantification, the positive staining cells and intensity were assessed. IHC scoring of HMGB1 was higher in the I/R kidney (I/R group: 8.857 ± 0.5948) when compared with the luteolin-treated rats (I/R + luteolin group: 6.429 ± 0.8123) or control group (control group: 0.6000 ± 0.2449). The immunostaining of NF-*κ*B was also significantly higher in the saline-treated kidneys (I/R group: 9.571 ± 0.5714) compared with the luteolin-treated kidneys (I/R + luteolin group: 6.857 ± 0.5948) and nonischemic controls (control group: 0.5000 ± 0.2887) (Figure 6(b)).

### 3.7. Luteolin Treatment Was Effective in Inhibiting Renal Cell Apoptosis

**Figure 7:**
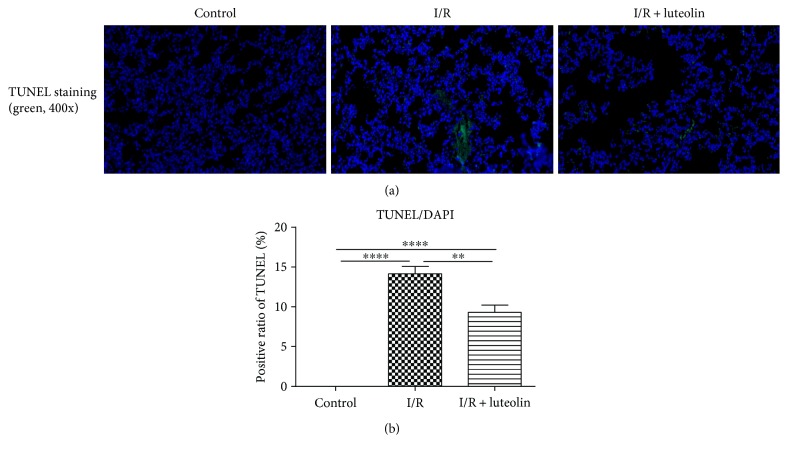
Luteolin treatment inhibited renal cell apoptosis in rats 24 hours after reperfusion. Renal tissues from control, I/R, and I/R + luteolin group rats were collected 24 hours after reperfusion. (a) Representative photographs of TUNEL staining (green) indicated apoptotic cells; (b) the positive ratios of TUNEL staining cells were counted. All data are expressed as the mean ± SEM, *n* = 6. ^∗∗^*P* < 0.01 and ^∗∗∗∗^*P* < 0.0001.

Kidneys from the I/R group showed major positive TUNEL staining, predominantly in the cortex and outer medulla. At 24 hours after experimentation, luteolin treatment had a significant effect on renal cell apoptosis. Kidneys from the luteolin-treated group had less TUNEL-positive staining (Figure 7(a)). The semiquantitation of TUNEL staining showed that the count of TUNEL-positive cells was remarkably reduced in the luteolin-treated group in comparison to the I/R rats after 24 hours of reperfusion (Figure 7(b)).

### 3.8. Luteolin Treatment Was Effective in Inhibiting ER Stress

**Figure 8:**
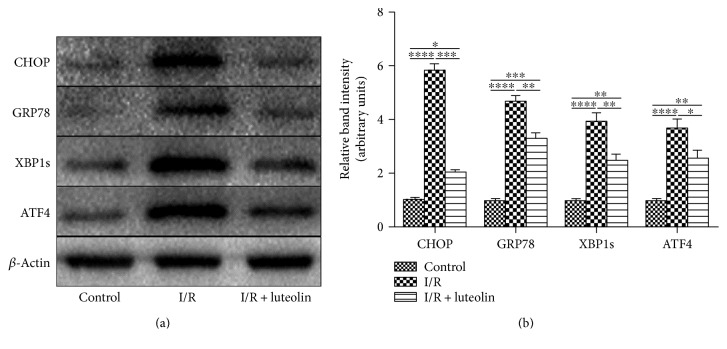
Luteolin treatment suppressed endoplasmic reticulum (ER) stress in rats 24 hours after reperfusion. Renal tissues of control, I/R, and I/R + luteolin group rats were collected 24 hours after reperfusion. (a) The protein levels of CHOP, GRP78, XBP1s, and ATF4 at 24 hours in renal tissues were shown as Western blot bands; (b) the relative band densities were calculated. All data are expressed as the mean ± SEM, *n* = 6. ^∗^*P* < 0.05, ^∗∗^*P* < 0.01, ^∗∗∗^*P* < 0.001, and ^∗∗∗∗^*P* < 0.0001.

Endoplasmic reticulum (ER) stress played an important role in the development of renal I/R injury. We tested the ER stress in the renal tissues by Western blot. Kidneys from the I/R group displayed high expression levels of CHOP, GRP78, XBP-1s, and ATF-4 when compared with the control and I/R + luteolin group. However, treatment with luteolin could significantly decrease the expression levels (Figure 8(a)). The relative band intensities of these proteins were calculated and the scoring showed the same results with the blotting (Figure 8(b)).

## 4. Discussion

**Figure 9:**
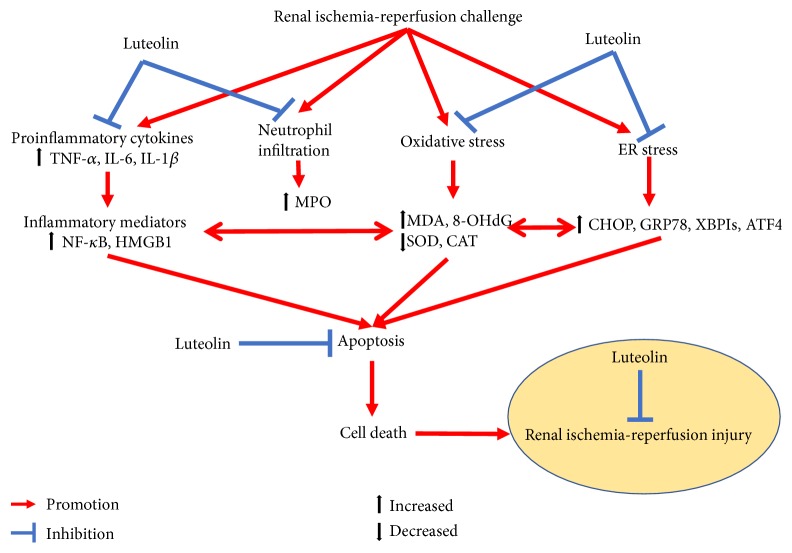
Schematic diagram of the protective role of luteolin in renal ischemia-reperfusion injury.

Treatment and mechanism-related studies of ischemia-reperfusion-induced renal injury remain popular in the development of kidney-related surgery and transplantation. The results of the present study showed that luteolin could reverse the renal dysfunction, histological damages of renal injury, oxidative stress, neutrophil accumulation, inflammatory reaction, apoptosis, and endoplasmic reticulum stress during renal I/R injury in rats. It could be concluded that anti-inflammation, antioxidative stress, antiapoptosis, and antiendoplasmic reticulum stress functions of luteolin might play key roles in mitigating the renal I/R injury (Figure 9).

Because of its special architectural features, the kidney is extremely sensitive to anoxia, which makes it vulnerable to hypoxic injury. Oxidative stress is considered the key step in the initiation and development of renal I/R injury [13]. For example, severe oxidative stress can make renal transplantation grafts very prone to acute and chronic rejection [14]. ROS are initially triggered by dysfunction of the mitochondrial respiratory chain in the ischemia phase and magnified in the reperfusion phase, which can cause cell death by directly impairing DNA, proteins, and lipids [15]. Enzymatic and nonenzymatic systems are the endogenous defenses for ROS [16]. However, excessive ROS production and reduction of antioxidant capacity results in the deterioration of I/R-induced renal injury [17]. MDA (bioproducts of lipid peroxidation) and 8-OHdG (bioproducts of oxidative DNA damage) are the classical indicators of oxidative stress. SOD and CAT, the endogenous antioxidants, are indirect markers of the ability of free radical generation. They are the “negative and positive” markers of the levels of oxidative stress [18]. Our study showed that luteolin significantly reduced the MDA and 8-OHdG levels and increased the SOD and TAC activities after renal I/R injury. Luteolin treatment might reduce the oxidative stress, increase the antioxidant capacity, and then decrease subsequent renal injury. Previous studies have showed that luteolin has great antioxidant effects. Yu et al. found that luteolin could decrease MDA levels and increase SOD levels in myocardial I/R injury [19]. Luteolin also exerted antioxidant abilities in acetaminophen-induced liver injury, cancer development, d-galactose-induced renal damage, and so forth [20–22].

Polymorphonuclear neutrophil infiltration is characteristic of acute injury induced by tissue ischemia-reperfusion, drug toxicity, shock, and so forth [23]. The migration and activation of neutrophils in the ischemic kidney will release ROS, MPO, inflammatory factors, and so forth which can promote and exacerbate the renal injury [24]. Linas et al. showed that activated neutrophils could significantly aggravate the renal I/R injury [25]. The results of the present study showed that luteolin treatment could significantly inhibit tissue neutrophil infiltration and MPO activity and protect the tissue against I/R injury. This was not the first discovery that luteolin could inhibit neutrophil infiltration. Kuo et al. found that luteolin suppressed infiltration of neutrophils and activation of MPO activity in mice with endotoxin-induced acute lung injury [26]. Domitrovic et al. also discovered that luteolin could decrease the MPO activity in CCL4-induced hepatotoxicity in mice [27]. The inhibition of ROS production by luteolin might contribute to reduce neutrophil infiltration because of the close relationship between neutrophil activation and ROS production.

The inflammatory response is an important pathophysiological process in I/R-induced renal injury [4]. Neutrophil infiltration, ROS production, and tubular epithelial cell activation all can trigger and exaggerate the inflammatory cascade through the innate and adaptive immune systems. In turn, proinflammatory cytokines, including TNF-*α*, IL-1*β*, and IL-6, can promote the localized tissue injury to remote injury through the neutrophil activation and infiltration [28]. The TNF-*α*, IL-1*β*, and IL-6 levels were increased in renal I/R injury in the majority of studies. TNF-*α* is the upstream molecule of the inflammatory cascade, which can initiate the upregulation of cytokines and chemokines. IL-1*β* and IL-6 are the downstream molecules in the inflammatory cascade, which can directly impair the renal cell [29]. HMGB1 is a proinflammatory cytokine that exerts its actions mainly through the receptors for RAGE and through TLRs [30]. Wu et al. found that endogenous HMGB1 contributed to renal I/R injury, and the administration of recombinant HMGB1 could provide significant protection [31, 32]. In turn, the augmentation of inflammatory responses by releasing cytokines can be regulated by NF-*κ*B, which is also an important therapeutic target [33]. In the study, the renal tissue levels of TNF-*α*, IL-1*β*, and IL-6 were significantly increased in renal I/R injury. However, luteolin treatment could decrease these cytokines to protect the kidney against I/R-induced renal injury. Moreover, the immunohistochemical staining results showed that luteolin treatment could also decrease the expression levels of NF-*κ*B and HMGB1. Luteolin has anti-inflammatory effects that involve the activation of the antioxidative enzyme system, the suppression of NF-*κ*B, and the inhibition of proinflammatory cytokine release. Seelinger et al. showed that TNF-*α*, IL-1*β*, IL-6, and NF-*κ*B were all targets of luteolin [34]. Chen et al. also found that luteolin could inhibit LPS-triggered secretion and relocation of HMGB1 in septic diseases [35].

Evidence shows that apoptosis also contributes to renal I/R damage [36]. The study found that luteolin administration significantly decreased the TUNEL-positive cells in the I/R rats. Our study suggested that the mitigation of I/R-induced renal injury presented by luteolin might involve the amelioration of apoptosis. The potential of luteolin in inhibiting apoptosis has been widely explored. Yu et al. found that luteolin could inhibit apoptosis in myocardial I/R injury [19]. Xin et al. also found that luteolin inhibited tubular apoptosis in lipopolysaccharide-induced acute renal injury in mice [9].

The endoplasmic reticulum is the subcellular organelle for protein folding and transporting, as well as for the biosynthesis of some lipids. Insults to the ER can lead to the accumulation of unfolded proteins in the ER and cause ER stress [37]. The process mainly involves hypoxia and nutritional deprivation during tissue I/R causing ER stress. In regard to renal I/R injury, renal tissues from patients, in vivo and in vitro experiments, showed that ER stress plays a key role in I/R-induced renal injury [38]. Our data suggested that inhibiting ER stress might contribute to the effect of luteolin on I/R-induced injury. Significant evidence has suggested that luteolin treatment could inhibit ER stress induced by physiological and pathological processes [20, 39, 40].

In conclusion, the present study found that luteolin treatment had protective capacity on I/R-induced renal injury in rats. The present study indicated that luteolin protected the kidney mainly by blocking ROS generation, inhibiting oxidative stress and increasing antioxidant ability, suppressing inflammation, decreasing cell apoptosis, and endoplasmic reticulum stress. Further studies should be performed to verify the safety and efficacy of luteolin in clinical applications.
